# Mechanical Fault Diagnosis of High-Voltage Circuit Breakers Based on Multi-Sensor Gramian Angular Field and Deep Residual Network

**DOI:** 10.3390/s26175604

**Published:** 2026-09-03

**Authors:** Xining Li, Hanyan Xiao, Ke Zhao, Lei Sun, Tianxin Zhuang, Haoyan Zhang, Hongwei Mei

**Affiliations:** 1 State Grid Jiangsu Electric Power Research Institute, Nanjing 211103, China; 1320211433@139.com (X.L.); 15950559834@163.com (H.X.); 15105168884@163.com (K.Z.); slsz_2014@126.com (L.S.); zhuangtx@js.sgcc.com.cn (T.Z.); 2 Shenzhen International Graduate School, Tsinghua University, Shenzhen 518055, China; haoyan-z24@mails.tsinghua.edu.cn

**Keywords:** high-voltage circuit breaker, mechanical fault diagnosis, multi-sensor fusion, Gramian Angular Field, Deep Residual Network

## Abstract

The mechanical reliability of high-voltage circuit breakers (HVCBs) is crucial for power-grid stability, yet traditional diagnostic methods rely heavily on manually extracted scalar features that can discard transient information. This paper presents a mechanism-aware diagnostic pipeline that combines synchronized coil-current, contact-travel, and spring-pressure measurements; Variational Mode Decomposition (VMD); kinematics-driven Region of Interest (ROI) alignment; Gramian Angular Field (GAF) encoding; RGB channel stacking; and ResNet-18 classification. On a controlled 220 kV experimental platform covering five operating conditions and 1500 operating-cycle samples, the framework achieved an average accuracy of 96.18% (macro-precision 96.10%, recall 96.01%, and F1-score 96.05%) under five repeated stratified 2:1 holdout evaluations. Single-channel controls obtained 88.47% for current, 90.24% for travel, and 85.13% for pressure; removal of VMD and ROI reduced accuracy to 94.72% and 93.46%, respectively. The results should be interpreted as proof-of-concept evidence on controlled simulated faults; validation on temporally separated field data, other breaker types and voltage levels, and naturally imbalanced fault distributions remains necessary before broad condition-based-maintenance deployment.

## 1. Introduction

With the rapid expansion of ultra-high voltage (UHV) power grids and the increasing complexity of modern power systems, the operational reliability of primary equipment faces unprecedented challenges. As the core protective and control equipment in substations, high-voltage circuit breakers (HVCBs) undertake the critical tasks of interrupting short-circuit currents and isolating faulty networks [1,2,3]. According to statistics from the International Council on Large Electric Systems (CIGRE), mechanical faults originating from the operating mechanism account for 40% to 50% of the total failures in circuit breakers, far exceeding electrical or insulation faults [3,4,5,6,7].

**Figure 1:**
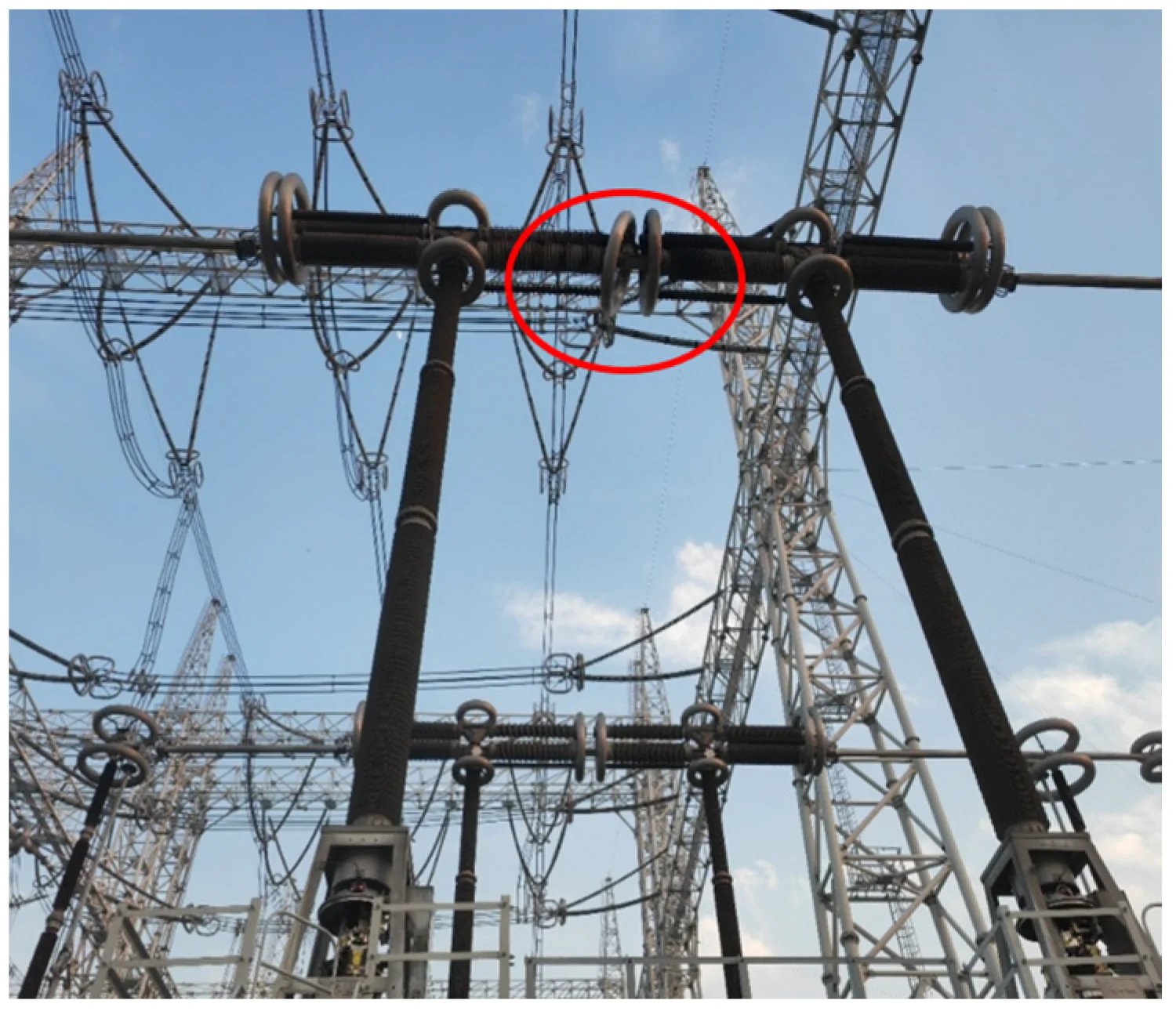
Closing failure of a phase-B circuit breaker in a converter station.

Spring operating mechanisms are widely utilized due to their compact structure and high output power. However, their operational characteristics of long-term static standby and instantaneous extreme stress release can easily induce latent defects such as spring fatigue, transmission chain jamming, and coil degradation [8,9,10,11]. Once these mechanical anomalies evolve into maloperation or refusal to operate, they can trigger cascading grid failures and massive economic losses [12,13,14]. Figure 1 illustrates a closing failure of a phase-B circuit breaker in a converter station. Therefore, achieving precise Condition-Based Maintenance (CBM) through advanced fault diagnosis technologies has become a consensus in the power industry.

Historically, HVCB fault diagnosis heavily relied on expert experience and threshold judgments based on 1D sensor data (such as coil current and vibration signals). With the rise of artificial intelligence, data-driven diagnostic approaches based on traditional Machine Learning (ML) algorithms (such as Support Vector Machines (SVMs), Random Forests (RFs), and K-Nearest Neighbors (KNN)) have been widely adopted [15,16,17,18,19]. Although these shallow models perform well in small-sample scenarios, they exhibit a notable limitation: heavy reliance on manual feature extraction [20,21,22].

Engineers must manually design and extract discrete scalar features from waveforms, such as maximum current, starting time, and static pressure values. This handcrafted dimensionality reduction forces continuous dynamic waveforms to collapse into fragmented single points. Consequently, the rich high-frequency microscopic distortion information—which is critical for early fault warning—is permanently lost, making these models vulnerable to novel or complex composite faults in noisy industrial environments.

Recently, 1D Convolutional Neural Networks (1D-CNNs) and Long Short-Term Memory (LSTM) networks have been introduced to automatically extract features from time-series sequences [23,24,25,26]. However, when dealing with multi-source heterogeneous signals spanning multiple physical domains, simply concatenating these 1D signals into a long vector severely disrupts their intrinsic physical synergy. The network struggles to effectively learn the cross-modal coupling mechanisms between the electrical trigger (coil), kinematic execution (travel), and dynamic power source (spring).

To overcome the limitations of manual feature construction and decouple heterogeneous physical signals, this paper introduces a time-series-to-image transformation strategy by transferring advanced Computer Vision (CV) techniques to the electrical engineering domain. The Gramian Angular Field (GAF) is a powerful mathematical tool that encodes 1D time-series data into 2D images, preserving absolute temporal dependencies in a polar coordinate system while capturing complex non-linear correlations [27,28,29,30].

By transforming HVCB waveforms into GAF images, the latent fault characteristics are explicitly visualized as diverse spatial textures (such as grid patterns, sharp edges, and smooth gradients). More importantly, mapping multi-source sensor signals into independent Red, Green, and Blue (RGB) color channels provides a three-channel representation compatible with Deep Residual Networks (ResNet). The organization of this paper is as follows: Section 2 describes the true-type experimental platform, the multi-source synchronous sensing matrix, and the simulated mechanical fault conditions. Section 3 details the proposed data processing methodology, including Variational Mode Decomposition (VMD) denoising, kinematics-driven smart ROI alignment, GAF-based 1D-to-2D mapping, and multi-sensor RGB image fusion. Section 4 presents the model training configuration, detailed fault classification performance, t-SNE feature space visualizations, and a comprehensive comparative analysis with ablation and baseline models. Finally, Section 5 concludes this paper and outlines potential avenues for future research.

Recent condition-monitoring research has expanded in three complementary directions. Physics-modeling-driven interpretable data augmentation has been used to generate physically plausible minority-fault samples under class imbalance [31]. Non-exemplar incremental prognostics based on a knowledge library network have been proposed to retain previously learned degradation knowledge without storing historical exemplars [32]. Time-series foundation models, including TimeGPT-based mechanical degradation assessment, further illustrate the potential of zero-shot inference and uncertainty-aware transfer across machines [33]. These advances motivate future HVCB systems that can cope with scarce faults, continual model updating, and cross-device transfer. The present study addresses a narrower problem: discriminating millisecond-scale breaker operating conditions from synchronized heterogeneous transient measurements.

The contribution is therefore not a claim that VMD, GAF, RGB stacking, or ResNet is individually new. Rather, it is the mechanism-aware integration of (i) a shared trigger and contact-travel-based ROI that aligns electrical excitation, kinematic execution, and spring-energy release; (ii) a fixed physical-to-color assignment that preserves sensor identity while enabling two-dimensional cross-channel convolution; and (iii) controlled single-sensor comparisons that quantify the complementarity of current, travel, and pressure. Compared with single-sensor GAF diagnosis, time-frequency imaging of current/displacement, and feature-level ensemble learning reported for HVCBs [34,35,36,37], the framework provides an image-level route for jointly representing three physically distinct stages of one operating cycle. Its practical value and limitations are discussed in Section 4.4, Section 4.5 and Section 4.6.

## 2. Experimental Platform and Fault Simulation

### 2.1. Sensors and Installation

**Figure 2:**
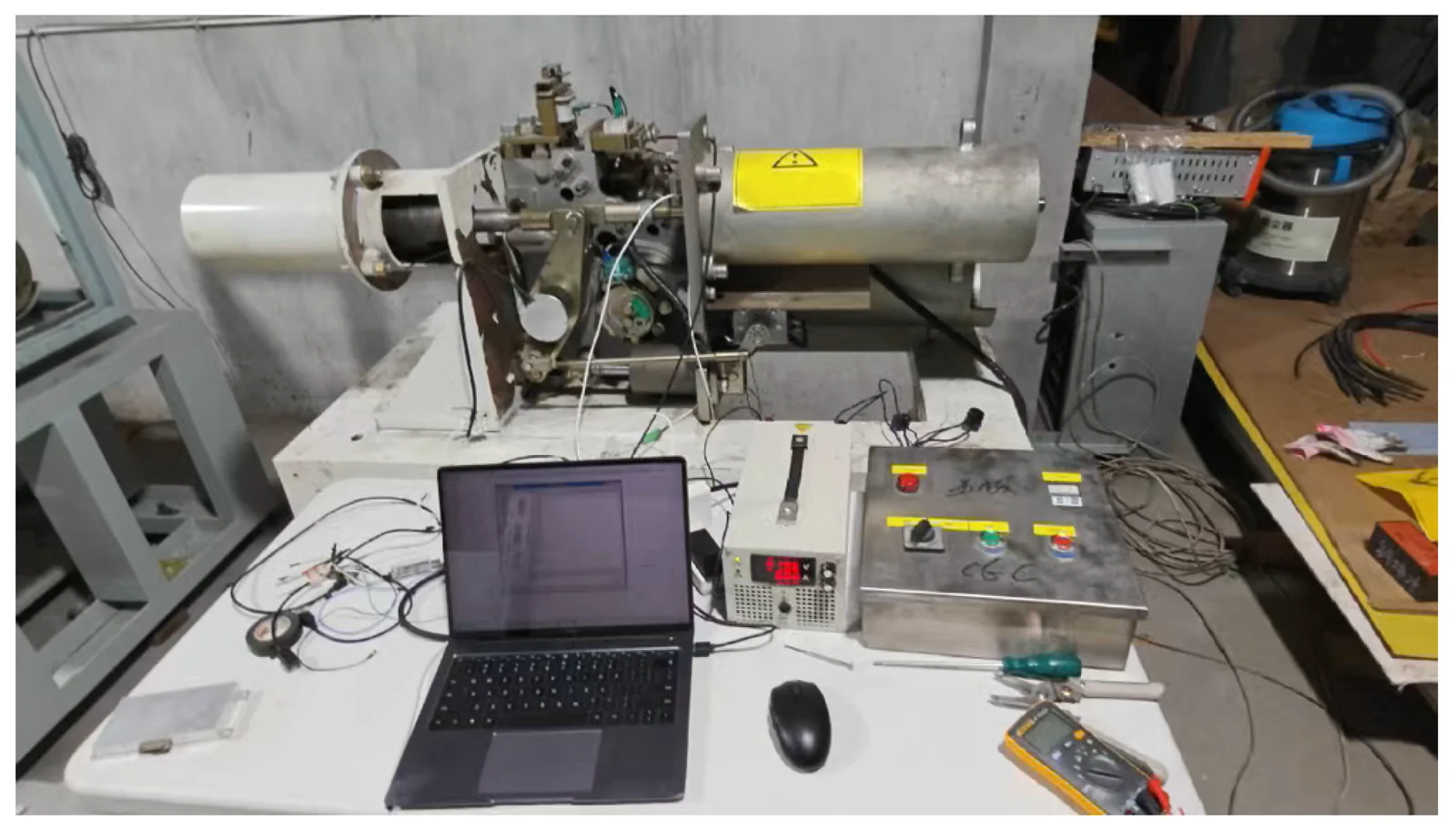
The 220 kV circuit breaker experimental platform.

To capture the intense transient mechanical and electrical variations in HVCBs within tens of milliseconds, a 220 kV spring operating mechanism experimental platform equipped with a multi-source high-frequency synchronous sensing matrix was constructed, as shown in Figure 2. Unlike traditional periodic maintenance testing that focuses on static parameter measurements, this system emphasizes the absolute time-domain alignment and continuous waveform tracking of dynamic operations.

**Figure 3:**
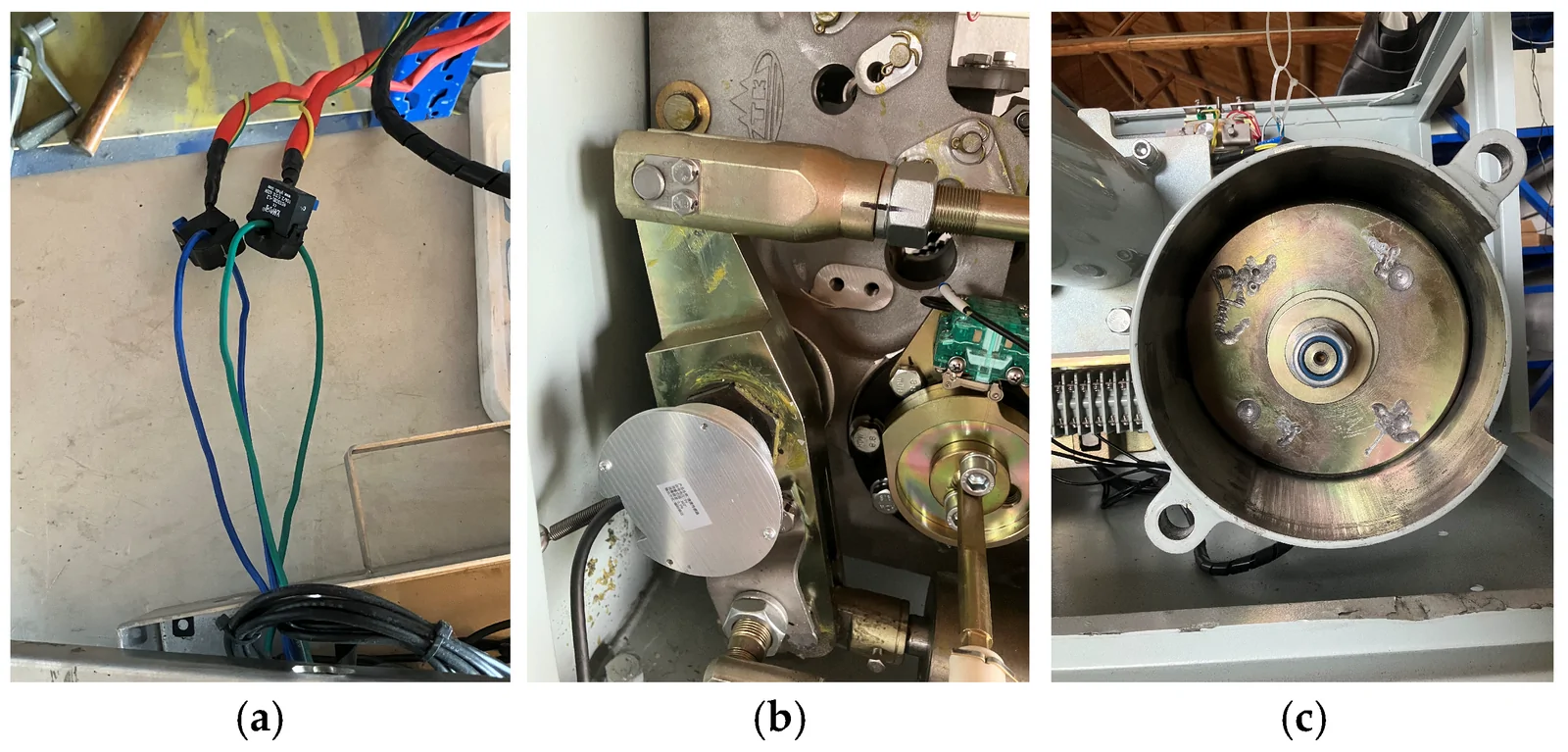
Sensing system: (**a**) spring pressure sensor; (**b**) opening/closing coil current sensor; (**c**) contact travel sensor.

The sensing matrix comprises three key physical dimensions:

**Electromagnetic Trigger (Coil Current):** High-precision Hall effect sensors are installed non-invasively to monitor the opening/closing coil current and the energy storage motor loop. This allows for continuous tracking of the control loop’s electromagnetic response without interfering with the original electrical wiring. The opening/closing coil current sensor is shown in Figure 3b.

**Kinematic Execution (Contact Travel):** A non-contact photoelectric encoder is flexibly mounted on the mechanism’s reserved main shaft hole via custom brackets. The flexible connection effectively eliminates the severe vibration interference caused by the shock of the mechanism, thereby obtaining an ultra-smooth continuous travel curve. The contact travel sensor is shown in Figure 3c.

**Dynamic Power (Spring Pressure):** Customized ring-type Micro-Electro-Mechanical System (MEMS) hydraulic pressure sensors are embedded coaxially between the operating springs and the end pressure plates. This configuration allows for real-time capturing of the high-frequency elastic potential energy release process during actions.

All heterogeneous sensor signals are synchronized by a multi-channel data acquisition unit with a unified clock source, ensuring strict phase consistency among current, travel, and pressure. This absolute spatiotemporal alignment is an essential prerequisite for the subsequent GAF cross-modal image fusion, guaranteeing that multi-physical domain features do not suffer from temporal misalignment. The pressure sensor is shown in Figure 3a.

For each operation, the acquisition record contained timestamped scalar sequences of coil current (A), moving-contact travel (mm), and spring pressure (kN), together with the condition label and cycle identifier. The current and travel channels were sampled at 10 kHz (100 μs interval), while the pressure channel was sampled at 200 kHz (5 μs interval). Each acquisition window lasted 100 ms. Accordingly, every raw record contained 1000 current samples, 1000 travel samples, and 20,000 pressure samples. The shared hardware trigger provided the common time origin. After ROI extraction, the three channels were resampled to a common length of 224 points before GAF encoding so that corresponding RGB pixels represented the same normalized phase of the operating event.

### 2.2. Fault Simulation and Waveform Distortion Mechanisms

**Figure 4:**
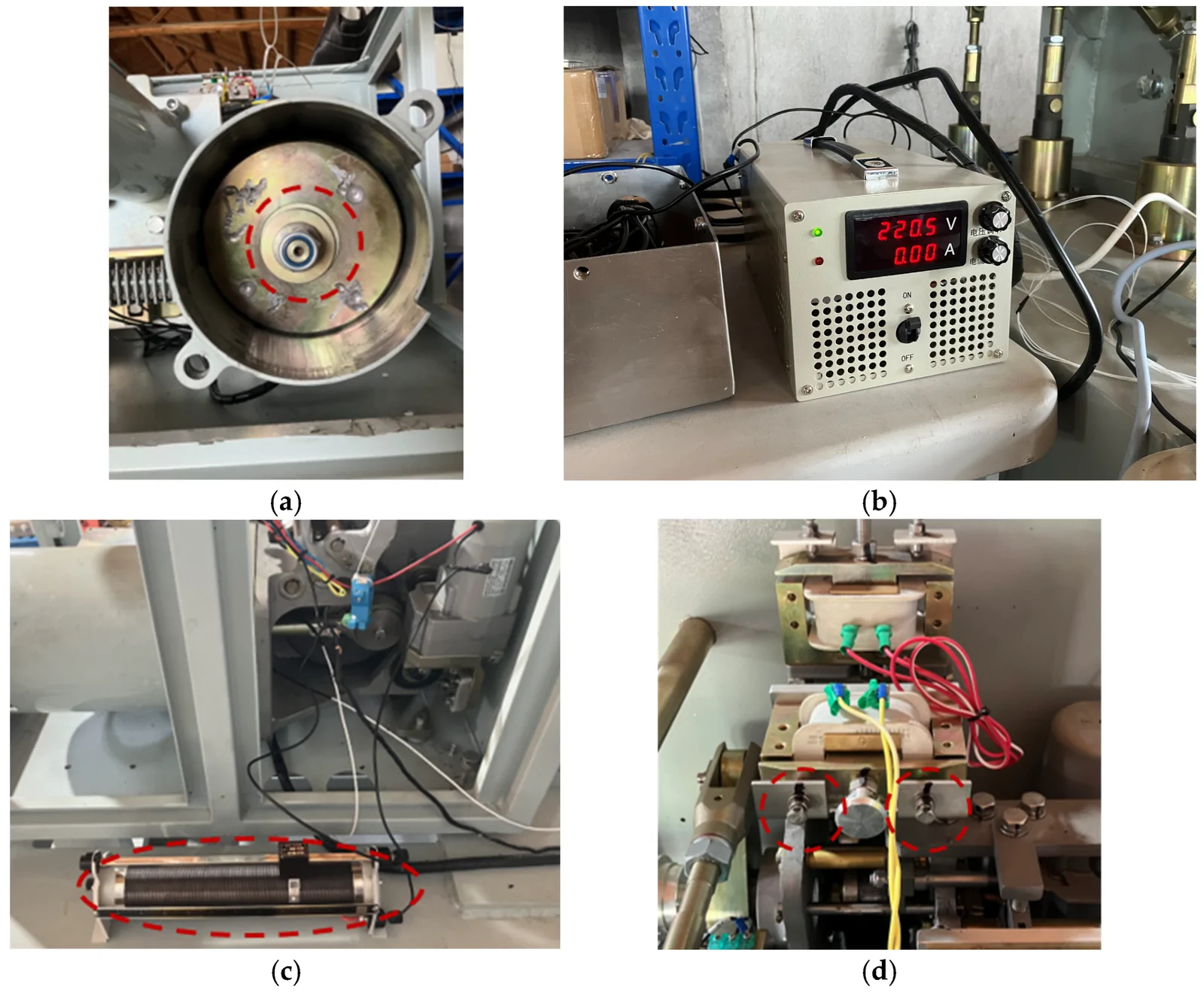
Fault simulation experiments: (**a**) DC power supply adjustment for coil degradation; (**b**) spring fatigue; (**c**) sliding rheostat for motor coil aging; (**d**) plunger adjustment for electromagnet core idle stroke.

To comprehensively evaluate the diagnostic framework, typical operational conditions were simulated on the 220 kV platform: normal state (S1), spring fatigue (S2), opening/closing coil degradation (S3), energy storage motor coil aging (S4), and electromagnet core idle stroke (S5). Instead of causing a simple static numerical change, these simulated mechanical faults change the waveform characteristics. The specific simulation methods and waveform distortion mechanisms of each fault are described as follows:

**Spring Fatigue (S2):** The spring compression bolt was loosened by 1 mm to simulate spring fatigue, as shown in Figure 4b. After the experiment, a specialized spring tool was installed to press the spring back to its initial position, verifying the exact mechanical reset via the pressure sensor’s reading. This spring relaxation reduces the stiffness of the mechanical chain. Reflected in the high-frequency continuous waveform, it leads to a noticeable deceleration in the travel slope (operating speed) and induces high-frequency oscillation glitches at the tail end of the pressure release curve.

**Opening/Closing Coil Degradation (S3):** An external DC power supply box was used to directly adjust the supply amplitude of the opening/closing coils, as shown in Figure 4a. The initial normal voltage of 220 V was reduced to a fault voltage of 198 V. This voltage deficiency flattens the rising edge of the coil current and reduces its peak, delaying the buildup of electromagnetic force. Consequently, it manifests in the time series as a significant backward phase shift in the entire subsequent mechanical action trajectory.

**Energy Storage Motor Coil Aging (S4):** A sliding rheostat of 50 Ω was connected in series in the energy storage motor coil loop to simulate the coil aging fault, as shown in Figure 4c. During operation, the rheostat was adjusted to insert a resistance of 5 Ω. The increased coil impedance decreases the output power, which stretches the motor current envelope along the time axis, accompanied by severe current fluctuations due to uneven torque.

**Electromagnet Core Idle Stroke (S5):** The electromagnet core idle stroke fault was simulated by loosening the core bolts on both sides by 1 mm, as shown in Figure 4d. This creates an excessive mechanical clearance before the plunger strikes the latch, which significantly increases the time delay between the electromagnetic trigger command and the actual kinematic execution. This anomaly leaves a distinct, prolonged “flat dead zone” in the early stage of the travel waveform.

The controlled fault-severity parameters were fixed at one level in the present dataset: 1 mm spring-bolt relaxation for S2, a reduction from 220 V to 198 V for S3, 5 Ω series resistance for S4, and 1 mm core-bolt loosening for S5. The 300 samples per condition were 300 distinct physical operating cycles acquired after the corresponding setting and reset procedure; they capture cycle-to-cycle variations in trigger timing, friction, sensor noise, and transient response, but they do not constitute a sweep over multiple fault severities, ambient temperatures, loads, or devices. This distinction is important when interpreting within-class diversity and engineering generalization.

These waveform distortions, phase shifts, and abnormal time delays uniquely imprint the physical anomalies into the continuous time-series data. Consequently, transforming these 1D dynamic waveforms into 2D GAF images provides a rich spatial representation for convolutional feature extraction and reduces dependence on manually selected scalar features.

## 3. Data Processing

**Figure 5:**
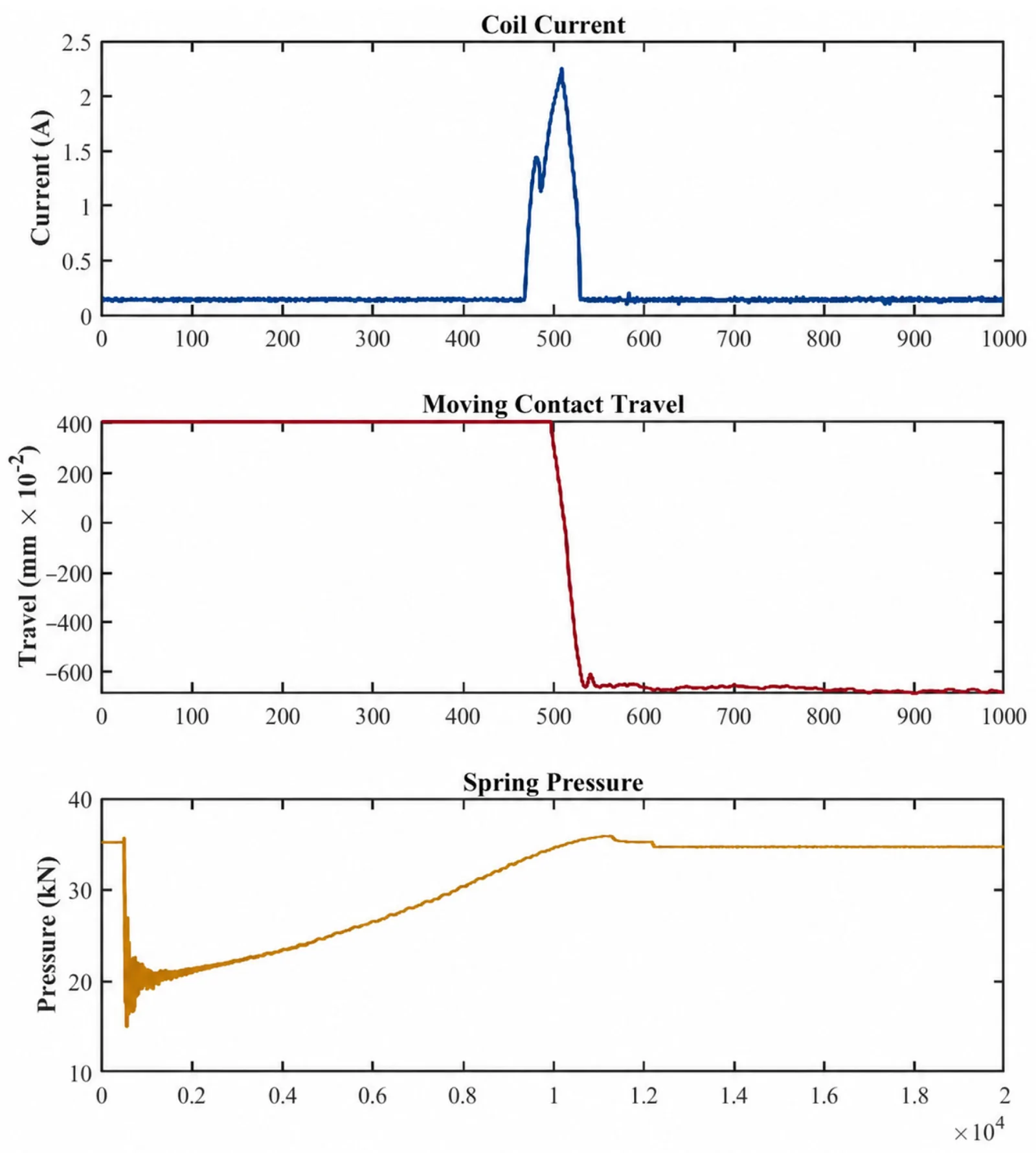
Aligned 1D multi-sensor waveforms.

The 1D transient waveforms of opening/closing current, moving contact travel, and spring pressure acquired by the sensors represent the critical physical states of the HVCB during its opening/closing operation, as illustrated in Figure 5.

### 3.1. Variational Mode Decomposition (VMD) Denoising and Smart Waveform Alignment

Due to the strong electromagnetic interference (EMI) in substation environments, the raw signals collected by sensors inevitably contain high-frequency noise. Conventional filters or basic wavelet transforms often suffer from fixed basis functions or mode mixing. To preserve the high-frequency abrupt edges of the HVCB waveforms while adaptively filtering out noise, Variational Mode Decomposition (VMD) is employed in this paper.

VMD is a non-recursive, entirely adaptive signal processing technique. It decomposes the original noisy signal x(t) into K discrete Band-Limited Intrinsic Mode Functions (BLIMFs) denoted as $u_{k}(t)$. The core logic of VMD is to formulate a constrained variational optimization problem, aiming to minimize the sum of the estimated bandwidths of all modes:

(1) $$min\sum ||\partial_{t}[(\delta (t)+j/(\pi t))\;*\;u_{k}(t)]exp(-j\omega_{k}t)|{|}^{2}$$

subject to the condition:

(2) $$\sum u_{k}(t)=x(t)$$

where $\omega_{k}$ represents the center frequency of the k-th mode, δ(t) is the Dirac distribution, and ∗ denotes the convolution operator. To render this constrained problem unconstrained, a quadratic penalty factor α and a Lagrangian multiplier λ(t) are introduced to construct the augmented Lagrangian function L:

(3) $$L=\alpha \sum ||\partial_{t}[(\delta (t)+j/(\pi t))\;*u_{k}(t)]exp(-j\omega_{k}t)|{|}^{2}\;+||x(t)-\sum u_{k}(t)|{|}^{2}+\langle \lambda (t),x(t)-\sum u_{k}(t)\rangle$$

The Alternating Direction Method of Multipliers (ADMM) is then applied to iteratively update $u_{k}$, $\omega_{k}$, and λ in the frequency domain. Upon convergence, the signal is decoupled. By calculating the Pearson correlation coefficient between each BLIMF and the original signal, modes dominated by high-frequency noise are discarded, and the remaining principal modes are reconstructed to obtain the denoised signal.

In implementation, the VMD mode numbers were set to K = 5 for coil current, K = 4 for contact travel, and K = 6 for spring pressure. For all three channels, the quadratic penalty factor was α = 2000, the maximum number of iterations was 500, and convergence was declared when the relative update error fell below 10^−7^. A decomposed mode was retained when the absolute value of its Pearson correlation coefficient with the corresponding raw signal satisfied |r| ≥ 0.20. These settings were held constant within each sensor channel across all evaluation runs and were determined using training data only.

**Figure 6:**
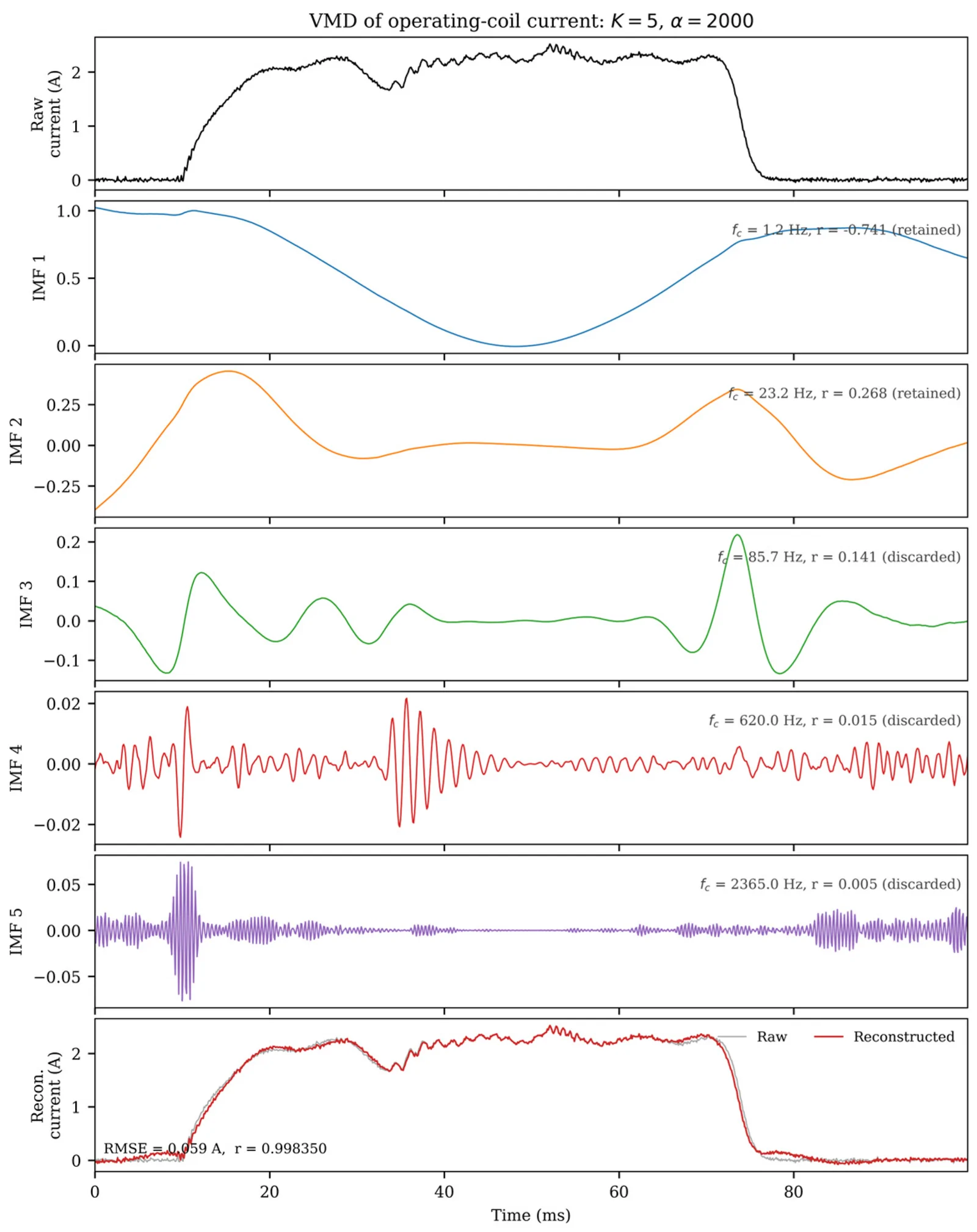
VMD and reconstruction of an S1 coil-current signal.

For the representative example in Figure 6, IMF1 and IMF2 satisfied the absolute Pearson threshold |r| ≥ 0.20 and were retained together with the slowly varying residual. The reconstructed signal achieved a correlation coefficient of 0.99835 with the original waveform and an RMSE of 0.059 A. At the pipeline level, the VMD-on/off comparison provides complementary classification evidence: removing VMD reduced the average accuracy from 96.18% to 94.72% under identical holdout splits.

After VMD denoising, the raw data consists of long sequences, where the actual mechanical action only occupies a small fraction of the time window. Feeding the entire sequence directly into the 2D transformation would result in large, featureless solid-color blocks in the generated image. Therefore, a kinematics-driven smart Region of Interest (ROI) alignment algorithm is proposed. By calculating the first-order derivative of the contact travel v(t)=dx(t)/dt, the exact starting point of the mechanical action is accurately locked when the velocity exceeds a predefined threshold. The waveform is then dynamically clipped to retain only the essential transient process, reducing the influence of inactive signal regions.

The action onset was defined as the first instant at which the absolute contact-travel velocity exceeded 5% of the peak absolute velocity of that operating cycle. The ROI retained 10 ms before the detected onset and 50 ms after it. Each clipped channel was resampled to 224 points using cubic-spline interpolation, independently normalized to [−1, 1], and then converted into a 224 × 224 GAF image. The onset rule, interpolation method, and normalization procedure did not use testing labels.

### 3.2. 1D to 2D Transformation via Gramian Angular Field

To leverage the powerful spatial feature extraction capabilities of Convolutional Neural Networks (CNNs), the 1D aligned time-series data must be transformed into 2D images. The Gramian Angular Field (GAF) is introduced to encode the 1D signals into a polar coordinate system, while preserving temporal dependencies.

First, the aligned time-series X is normalized to the interval [−1,1]:

(4) $$X_{norm}=[X-min(X)]/[max(X)-min(X)]\times 2-1$$

Then, the normalized data is converted to polar coordinates, where the numerical values correspond to the angle ϕ:

(5) $$\phi_{i}=arccos(X_{norm,i})$$

Finally, the Gramian Angular Summation Field (GASF) matrix G is generated by calculating the cosine of the sum of angles for pairwise points:

(6) $$G_{i,j}=cos(\phi_{i}+\phi_{j})=X_{i}X_{j}-\sqrt{1-X_{i}^{2}}\sqrt{1-X_{j}^{2}}$$

This transformation preserves the temporal dependency of the original 1D signal on the diagonal and explicitly maps the dynamic waveform distortions into 2D spatial textures (e.g., grids and sharp edges).

### 3.3. Multi-Sensor RGB Image Fusion

HVCB fault diagnosis requires the collaborative analysis of multiple physical domains (electrical, kinematic, and dynamic). Direct concatenation of 1D signals may not fully capture their cross-modal relationships. Therefore, a physical-to-color channel mapping mechanism is proposed.

The GAF matrices of the three core sensors are independently mapped to the Red (R), Green (G), and Blue (B) channels of a standard color image:

R Channel (Closing Current): Captures the electromagnetic trigger and its rapid transient mutations, rendered as sharp crosshair textures in the fused image.

G Channel (Contact Travel): Reflects the continuous mechanical kinematic motion, mapped into classic checkerboard patterns.

B Channel (Spring Pressure): Represents the non-linear release process of elastic potential energy, presented as smooth color gradients.

**Figure 7:**
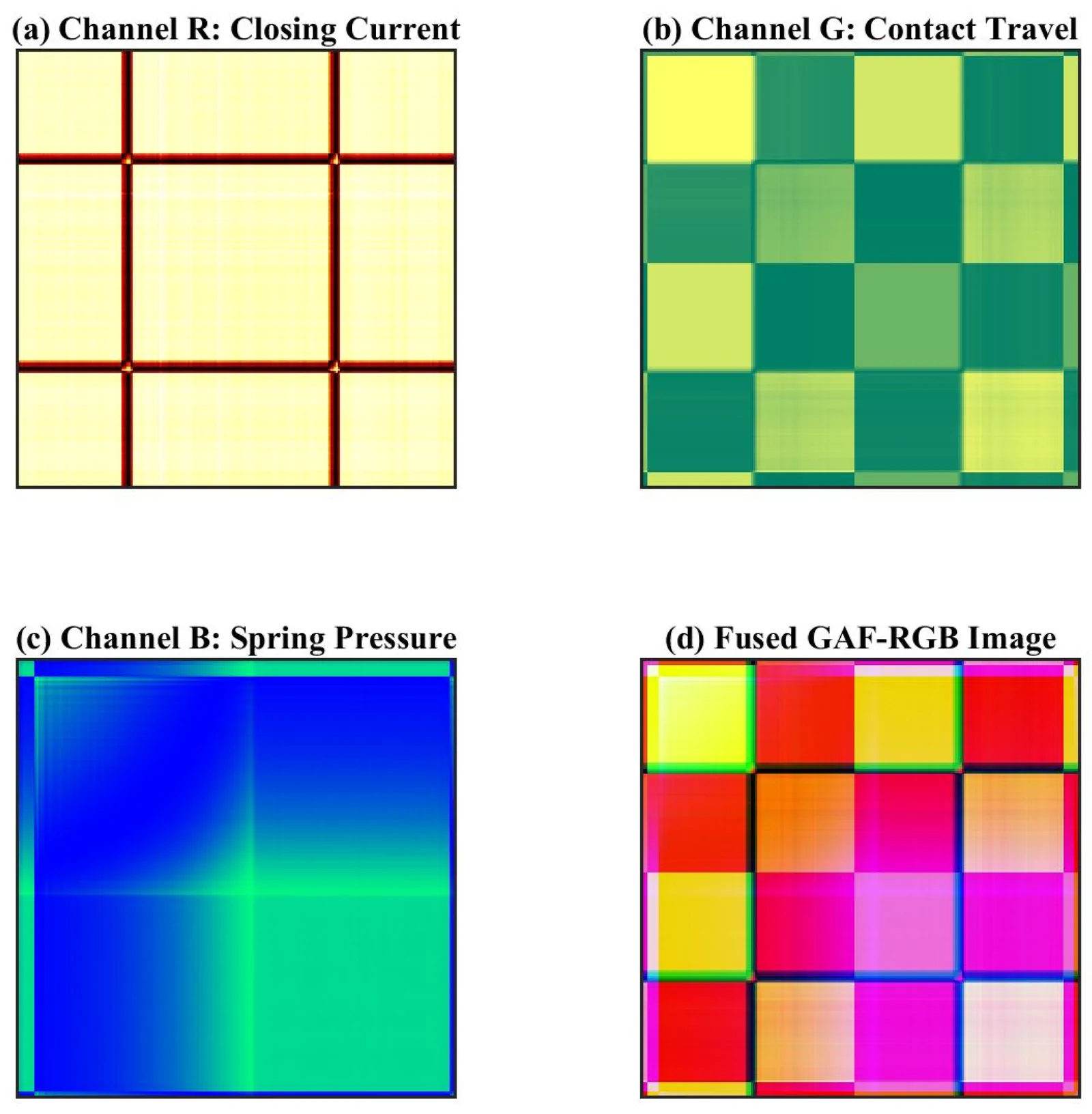
Multi-sensor GAF-RGB image fusion process: (**a**) Channel R (closing current); (**b**) Channel G (contact travel); (**c**) Channel B (spring pressure); (**d**) fused GAF-RGB image.

By stacking the three matrices pixelwise, I_RGB(i,j) = [G_current(i,j), G_travel(i,j), G_pressure(i,j)], a true-color feature image resized to 224 × 224 pixels is obtained, as shown in Figure 7. The channel assignment is fixed for every sample and evaluation run; therefore, color differences reflect changes in the relative temporal-correlation structures of the three physical domains rather than arbitrary pseudocoloring. The non-fused controls retain only one of these matrices and replicate it as a grayscale three-channel input to the same ResNet-18 backbone, ensuring that the performance difference is attributable to sensor fusion rather than network capacity. Figure 7 is retained as a compact construction example, while the class-level separability and within-class compactness are evaluated using the confusion matrix and the feature-space visualization.

## 4. Results and Discussion

### 4.1. Model Training and Hyperparameter Settings

To rigorously evaluate the proposed fault diagnosis framework, the multi-sensor GAF-RGB dataset was constructed utilizing the experimental data acquired from the 220 kV HVCB platform described in Section 2. For each of the five typical operating conditions—normal state (S1), spring fatigue (S2), opening/closing coil aging (S3), energy storage motor coil aging (S4), and electromagnet core idle stroke (S5)—exactly 300 dynamic operating cycles were executed, yielding a comprehensive dataset of 1500 samples in total.

Each physical operation produced one sample, and no fragments from a single cycle were distributed across training and testing subsets. In each of five repeated stratified holdout runs, 200 cycles per condition (1000 samples) were used for training and 100 cycles per condition (500 samples) for testing, corresponding to a 2:1 train-test ratio with balanced class proportions. The run-level metrics were then averaged. Because all cycles came from one platform and were acquired in repeated sessions, cycle-level separation prevents duplicate-window leakage but does not eliminate possible temporal or session correlation. A stronger future protocol should reserve complete acquisition sessions, devices, or sites as external test groups.

The proposed framework was implemented using PyTorch 2.1.0 on an industrial workstation, and the detailed training hyperparameters are summarized in Table 1.

**Figure 8:**
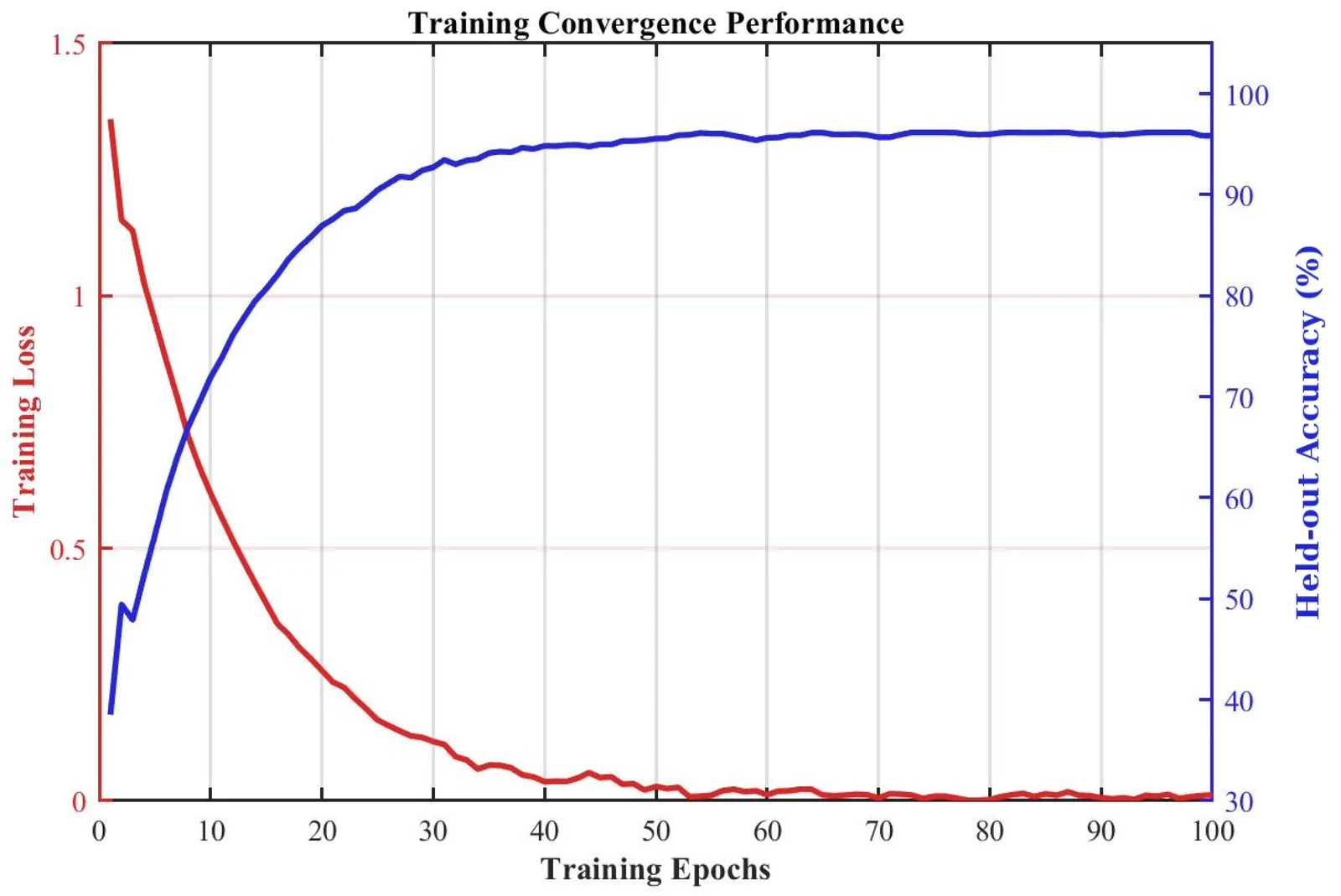
Training loss and held-out accuracy convergence curves of the proposed model.

During training, the loss and held-out accuracy were tracked across 100 epochs. As shown in Figure 8, the training loss decreased rapidly during the first 25 epochs and stabilized near 0.005 by epoch 40, while accuracy reached a plateau of approximately 96.18% after epoch 45. All models were trained for the fixed 100 epochs; the held-out curve was reported descriptively and was not used for epoch selection. These curves indicate stable optimization under the complete pipeline, but they do not by themselves isolate the effects of VMD or ROI alignment. Accordingly, the dedicated preprocessing ablations reported in Section 4.4 are used for component-level attribution.

### 4.2. Fault Classification Performance

The diagnostic performance of the proposed GAF-RGB ResNet framework was quantitatively evaluated on the held-out test subsets. To provide a comprehensive assessment, three standard statistical metrics—Precision (P), Recall (R), and F1-score ($F_{1}$)—were calculated for each operational condition. The corresponding mathematical formulations are defined as follows:

(7) $$Precision=\frac{TP}{TP+FP}$$

(8) $$Recall=\frac{TP}{TP+FN}$$

(9) $$F_{1}-score=\frac{2\times Precision\times Recall}{Precision+Recall}$$

where TP, FP, and FN represent the number of true positives, false positives, and false negatives, respectively. The average macro-classification metrics across the five repeated holdout runs are detailed in Table 2.

**Figure 9:**
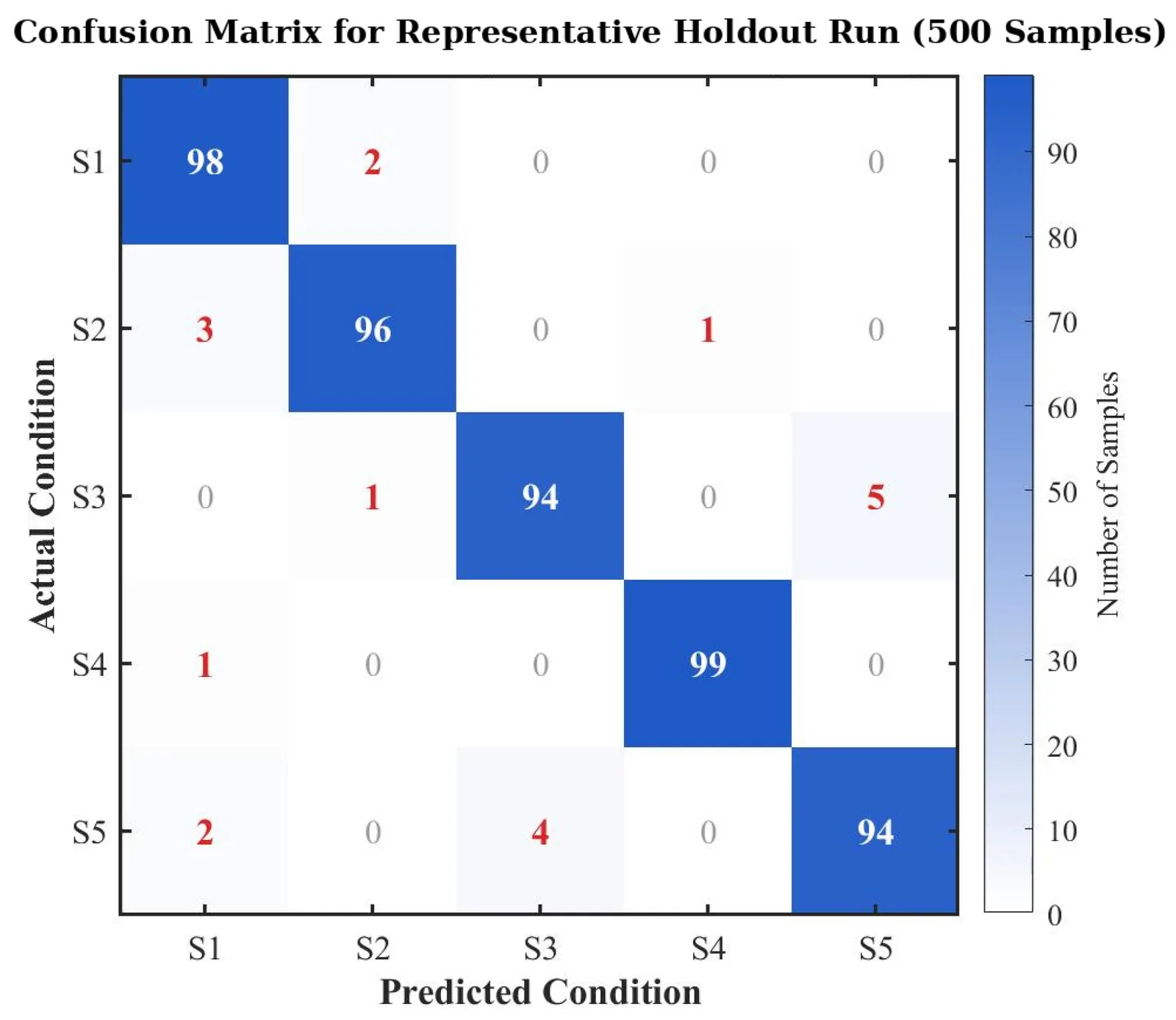
Confusion matrix of the representative holdout run on the held-out test subset.

To further illustrate the detailed classification accuracy, the confusion matrix of a representative holdout run on the held-out test subset (comprising 500 test samples, with 100 samples per condition) is plotted in Figure 9. For this representative holdout run, the proposed method achieved 96.20% (481/500 correctly classified).

A total of 19 samples were misclassified in this representative holdout run, which is consistent with the physical characteristics of the operating mechanism. Specifically, 3 samples of S2 (Spring Fatigue) and 2 samples of S5 (Idle Stroke) were misclassified as S1 (Normal State) because slight spring fatigue (1 mm loosening) and micro-clearances in their initial stages produce dynamic waveforms that are extremely close to the normal state, making them a well-known bottleneck in threshold-based detection. Additionally, 5 samples of S3 (Coil Aging) were misclassified as S5 (Idle Stroke), and 4 samples of S5 were misclassified as S3. This cross-misclassification is physically reasonable as both coil degradation and excessive plunger clearance delay the initiation of kinematic travel, creating similar temporal phase shifts. Nevertheless, the high overall macro F1-score of 96.05% and average accuracy of 96.18% verify that the spatial texture features mapped by GAF-RGB contain sufficient unique physical signatures.

### 4.3. Feature Visualization via t-SNE

**Figure 10:**
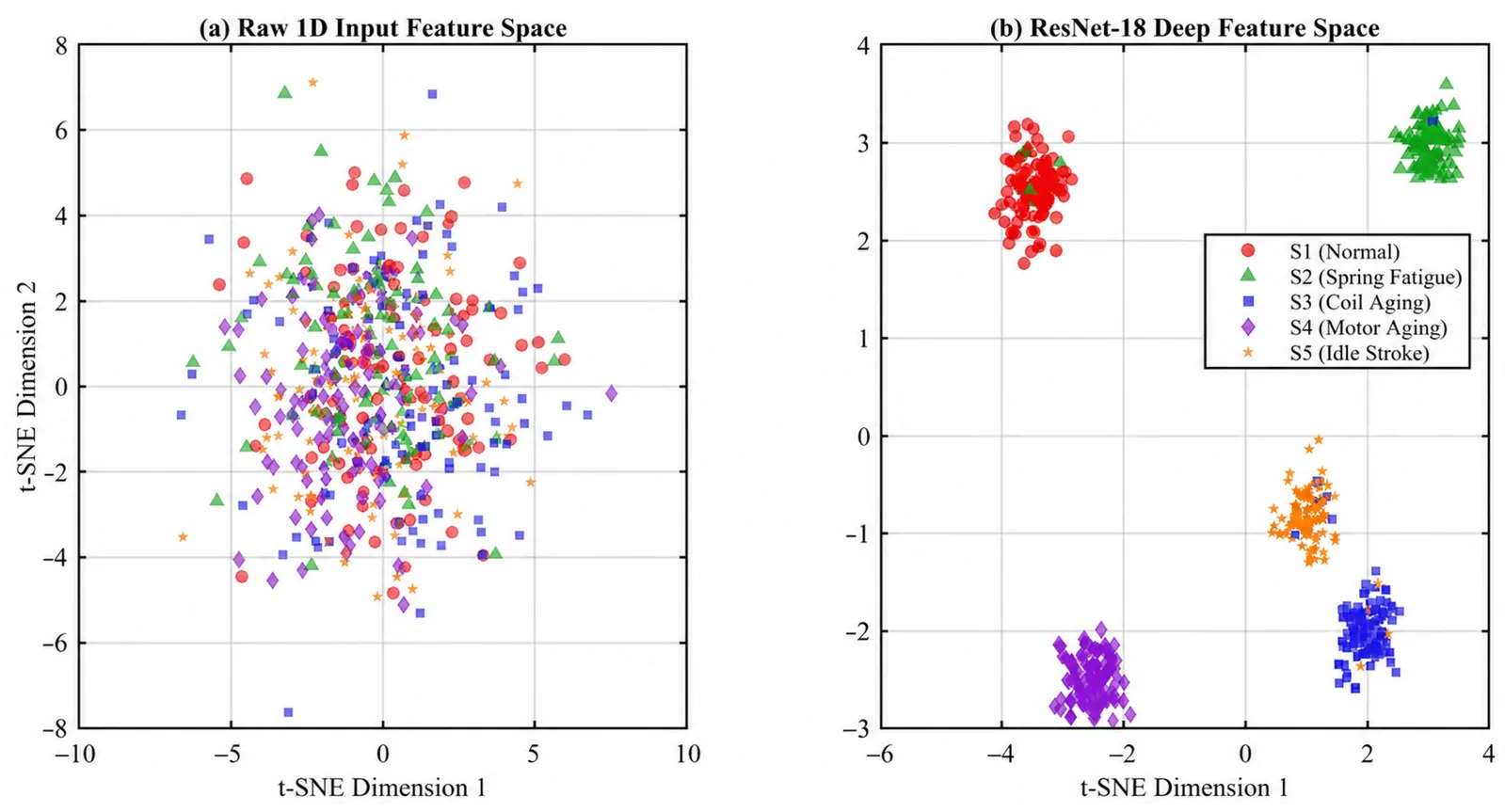
t-SNE feature space visualization: (**a**) raw 1D input space; (**b**) ResNet-18 deep feature space.

To visually interpret the high-dimensional feature representation extracted by the deep network and verify its decoupling capability, t-Distributed Stochastic Neighbor Embedding (t-SNE) was utilized. The feature distributions in the raw 1D input space and the deep spatial feature space (extracted from the average pooling layer of ResNet-18 before the fully connected layer) are projected into a 2D space, as qualitatively illustrated in Figure 10.

In the raw 1D input space (see Figure 10a), the 500 sample points of different fault conditions are heavily overlapped and scattered chaotically. This is because high-frequency noise and slight phase shifts obscure the boundaries between heterogeneous physical signals in the 1D time domain, indicating substantial overlap in this two-dimensional projection.

By comparison, in the ResNet-18 feature space (see Figure 10b), the sample points belong to five distinct, tightly clustered groups. The intra-class distance is reduced, while the inter-class boundaries are separated by wider margins. The transition points near cluster boundaries qualitatively mirror the confusion-matrix error pairs. This clustering pattern indicates that, for the reported dataset and projection, the GAF-RGB pipeline yields more separable feature representations than the raw 1D input visualization; it should not be interpreted as proof of general separability beyond the evaluated platform.

### 4.4. Ablation and Comparative Analysis

To examine the contribution of each preprocessing and representation component, all ablation and baseline models were evaluated using the same cycle-level holdout splits, class distributions, and training/test partitions. The comparisons include VMD and ROI removal, raw 1D input, STFT and CWT time-frequency images, individual-sensor GAF inputs, alternative 1D sequence models, an alternative 2D backbone, and handcrafted-feature classifiers. The average overall accuracies across the five repeated holdout runs are summarized in Table 3.

1. Evaluation of Dimension-Conversion (vs. 1D-CNN): A standard 1D-CNN was trained directly on the raw, concatenated 1D multi-sensor signals.
2. Evaluation of Multi-Sensor Channel Fusion (vs. Single-Channel GAF): Three independent ResNet-18 models were trained using GAF images from only one physical domain: current-only (red), travel-only (green), and pressure-only (blue).
3. Evaluation of Feature Extraction (vs. Shallow Machine Learning): Traditional Support Vector Machine (SVM) and Random Forest (RF) models were trained using manually designed hand-crafted features.

The average overall diagnostic accuracies across the five repeated holdout runs are compiled in Table 3.

The comparison results in Table 3 lead to several key conclusions:

Effect of 1D-to-2D Conversion: The proposed method outperformed the 1D-CNN by 4.87%. This highlights that converting 1D waveforms into 2D GAF images preserves the complex temporal correlations and phase dependencies.

Effect of Multi-Sensor Image Fusion: The non-fused Current-GAF, Travel-GAF, and Pressure-GAF controls achieved 88.47%, 90.24%, and 85.13%, respectively, under the same ResNet-18 backbone and data splits, whereas the fused RGB representation achieved 96.18%. The gains were 7.71, 5.94, and 11.05 percentage points over the R-, G-, and B-source controls, respectively. This supports complementary information across electromagnetic trigger, kinematic execution, and spring-energy release; it does not prove that RGB stacking is universally superior to all feature- or decision-level fusion methods.

Effect of VMD, ROI Alignment, and Representation: Removing VMD reduced accuracy from 96.18% to 94.72% (−1.46 percentage points), while removing ROI alignment reduced it to 93.46% (−2.72 points). Removing both components yielded 91.85%, confirming that their contributions are complementary under the reported protocol. CWT-ResNet-18 and STFT-ResNet-18 achieved 93.88% and 93.24%, respectively, whereas 1D-ResNet and TCN achieved 92.76% and 93.15%. The GAF-RGB EfficientNet-B0 control reached 95.37%, 0.81 points below the ResNet-18 configuration. These comparisons support the selected preprocessing and representation for this dataset without implying universal superiority across devices or datasets.

Comparison with Handcrafted-Feature Baselines: Traditional ML models using hand-crafted features achieved the lowest accuracies (83.59% and 81.16%), indicating lower performance of the evaluated handcrafted-feature baselines under the reported protocol.

### 4.5. Comparison with Recent HVCB Diagnostic Studies

Table 4 places the present framework alongside recent HVCB studies. The reported accuracies are descriptive only because the studies use different sensors, fault definitions, sample sizes, splits, and hardware. This literature-level comparison cannot replace re-implementation under a common protocol, but it clarifies the methodological distinction and the scope of the evidence.

### 4.6. Prior-Shift Robustness, Error Mechanisms, and Limitations

Operational datasets are normally dominated by healthy cycles and may also be imbalanced among fault types. As a limited prior-shift stress test, the class-conditional recalls from Table 2 were reweighted without retraining the balanced-data classifier. With an 80% normal/5% each-fault test prior, the expected accuracy is 97.58%; with a 60% normal/20% S2/10% S3/7% S4/3% S5 prior, it is 97.06%. These values show that the fixed classifier’s measured class sensitivities do not collapse under the two assumed test priors. They do not demonstrate robust learning from an imbalanced training set, because no imbalanced retraining, calibration, or minority-class augmentation experiment was conducted. Physics-informed augmentation and class-balanced/focal objectives are therefore important future extensions [31].

The 19 errors in the representative holdout run concentrate near physically transitional boundaries. S2 and S5 samples misclassified as S1 have small deviations in travel slope, onset delay, and pressure-release texture, so their fused GAF images retain large smooth regions similar to the normal class. The reciprocal S3-S5 errors arise because undervoltage and excess core clearance both delay travel onset; in the original domain this appears as a comparable phase shift between current and travel, while in the GAF domain it produces similar displaced edge and checkerboard intersections. By contrast, S4 remains well separated because the stretched and fluctuating motor-current envelope creates a more distinctive channel texture. These mechanisms suggest that future models should explicitly emphasize cross-channel lag and uncertainty near class boundaries rather than relying only on global texture similarity.

The evidence is limited to one 220 kV platform, one controlled severity per fault, repeated operating cycles, and simulated rather than naturally occurring field faults. Although one sample corresponds to one cycle, acquisition-session correlation may remain. The current results therefore support feasibility under controlled conditions, not universal deployment. External validation should use temporally separated sessions, multiple devices and voltage levels, naturally imbalanced field records, compound faults, open-set conditions, calibrated uncertainty, and device/session-level holdout splits.

## 5. Conclusions and Future Work

### 5.1. Conclusions

In this study, a visual–spatial fault diagnosis framework for high-voltage circuit breakers (HVCBs) was investigated using synchronized GAF-RGB representations and a Deep Residual Network (ResNet). By acquiring high-frequency synchronized transient waveforms of closing current, contact travel, and spring pressure, and validating the framework systematically under five simulated operational conditions on a 220 kV true-type experimental platform, the diagnostic capability and physical interpretability of the proposed method were evaluated. The main conclusions drawn from this research are structured as follows:

Information Preservation via 1D-to-2D GAF Encoding: By encoding 1D transient sensor waveforms into 2D polar matrices via GAF, temporal dependencies are mapped into spatial texture correlations. This mapping preserves the continuous spatiotemporal topology of the transient physical signals. High-frequency microscopic distortions—such as travel slope deceleration, coil current phase-shifts, and tail-end spring pressure oscillations—are preserved, avoiding the information loss associated with conventional handcrafted scalar feature extraction.

Cross-Modal Parameter Integration via RGB Channel Stacking: Mapping the GAF matrices of closing current (R channel), contact travel (G channel), and spring pressure (B channel) into standard RGB color space offers an effective mechanism to integrate heterogeneous parameters. This configuration aligns multi-physical domain features with deep convolutional receptive fields, allowing the ResNet-18 network to simultaneously capture cross-modal coupling mechanisms between the control, kinematic execution, and dynamic power subsystems.

Statistical Diagnostic Accuracy and Sensitivity: Under the five repeated stratified 2:1 holdout evaluations, the proposed framework achieved an average macro-precision of 96.10%, recall of 96.01%, F1-score of 96.05%, and overall accuracy of 96.18%. The quantitative results and confusion-matrix analysis indicate high sensitivity on this controlled dataset, while also revealing persistent cross-classification between S3 (coil aging) and S5 (idle stroke) because of similar early-stage temporal phase shifts. These results require external validation before broader engineering generalization.

Feature-Space Visualization: The t-SNE projection provides a qualitative view of the learned representations. Relative to the overlapping raw 1D projection, the reported ResNet-18 feature projection contains more compact class clusters and clearer boundaries. Because t-SNE is a nonlinear visualization rather than a quantitative validation test, this figure is treated as supporting interpretation and not as proof of general feature separability.

In summary, mapping synchronized multi-sensor transient waveforms into color-coded GAF structures is a feasible proof-of-concept alternative to manual scalar-feature pipelines on the controlled dataset studied here. The component ablations and consistently split baseline comparisons support the selected VMD, ROI, GAF-RGB, and ResNet-18 configuration. The results do not yet establish field-wide condition-based-maintenance readiness, which still requires external device- and session-level validation together with naturally imbalanced and compound-fault data.

### 5.2. Future Work

Despite the satisfactory diagnostic performance demonstrated by the proposed framework, several directions remain for future research.

Lightweight Deployment on Embedded Edge Devices: To transition the proposed algorithm from industrial workstations to substation field applications, future work will focus on compressing the ResNet backbone network using knowledge distillation and network pruning, facilitating its deployment on low-power edge-computing platforms for real-time online monitoring.

Diagnosis of Compound and Simultaneous Faults: The current research assumed single-source independent faults. In practical operational environments, compound faults (e.g., simultaneous spring fatigue and coil degradation) may occur. Developing multi-label classification networks to resolve co-occurring mechanical anomalies represents a key objective.

Cross-Voltage Class and Transfer Learning Scenarios: Future research will explore the transferability of the trained GAF-RGB ResNet model across different voltage levels (e.g., from 220 kV platforms to 110 kV or 500 kV HVCBs) utilizing domain adaptation and transfer learning techniques to minimize data acquisition costs in engineering fields.

Imbalanced, Incremental, and Foundation-Model Learning: Future work will investigate physics-informed minority-class generation [31], non-exemplar incremental updating [32], and large time-series models with uncertainty estimation [33]. These directions may reduce dependence on balanced laboratory datasets while enabling continual adaptation to device aging and new breaker types.

## Figures and Tables

**Table 1:** Deep Residual Network training hyperparameter configuration.

Hyperparameter	Configuration Value
Backbone Architecture	ResNet-18
Optimizer	Adam
Initial Learning Rate	$10^{-3}$
Weight Decay	$10^{-4}$
Batch Size	32
Loss Function	Cross-Entropy Loss
Training Epochs	100

**Table 2:** Detailed diagnostic performance metrics for the five simulated conditions (mean of five repeated stratified holdout runs).

Condition ID	Precision (%)	Recall (%)	F1-Score (%)	Accuracy (%)
S1	95.62	98.11	96.85	-
S2	97.35	95.24	96.28	-
S3	93.89	94.12	94.01	-
S4	98.74	99.01	98.87	-
S5	94.92	93.56	94.24	-
Overall	96.10	96.01	96.05	96.18

**Table 3:** Overall diagnostic accuracy comparisons with ablation and baseline models (mean of five repeated stratified holdout runs).

Model Group	Diagnostic Architecture	Overall Accuracy (%)
Proposed	GAF-RGB ResNet-18 (Ours)	96.18
Ablation	Current-GAF ResNet-18	88.47
Ablation	Travel-GAF ResNet-18	90.24
Ablation	Pressure-GAF ResNet-18	85.13
Baseline	1D-CNN	91.31
Baseline	Support Vector Machine (SVM)	83.59
Baseline	Random Forest (RF)	81.16
Ablation	Without VMD	94.72
Ablation	Without ROI alignment	93.46
Ablation	Without VMD and ROI	91.85
Baseline	CWT image + ResNet-18	93.88
Baseline	STFT image + ResNet-18	93.24
Baseline	1D-ResNet	92.76
Baseline	Temporal Convolutional Network (TCN)	93.15
Baseline	GAF-RGB + EfficientNet-B0	95.37

**Table 4:** Descriptive comparison with recent HVCB diagnostic approaches.

Study	Signals	Representation/Model	Scope	Reported Accuracy
Yang et al. [34]	Vibration	GASF+GADF dual-channel CNN	220 kV; six conditions	99.02%
Shao et al. [35]	Current + displacement	Morlet images + ResNet-50	Composite mechanism faults	91.67%
Wang et al. [36]	Multiple mechanism sensors	Statistical features + RF-AdaBoost	Spring circuit breaker	Study-specific
Li et al. [37]	Pressure, travel, coil and motor current	Handcrafted features + stacking ensemble	220 kV; five conditions	96.10%
This study	Current + travel + pressure	GAF-RGB + ResNet-18	220 kV; five conditions	96.18%

Note: Values are reproduced from the cited studies and are not directly rank-comparable across datasets.

## Data Availability

The data presented in this study are available on request from the first author.
